# Four main therapeutic keys for Parkinson’s disease: A mini review

**DOI:** 10.22038/ijbms.2019.33659.8025

**Published:** 2019-07

**Authors:** Daniel Hernandez-Baltazar, Rasajna Nadella, Laura Mireya Zavala-Flores, Christian de Jesús Rosas-Jarquin, María de Jesús Rovirosa-Hernandez, Arnulfo Villanueva-Olivo

**Affiliations:** 1 CONACYT-Instituto de Neuroetologia, Universidad Veracruzana, Xalapa, Veracruz, Mexico; 2 IIIT Srikakulam, Rajiv Gandhi University of Knowledge Technologies (RGUKT); International collaboration ID:1840; India; 3 Centro de Investigacion Biomedica del Noreste. IMSS. Monterrey, Nuevo Leon. Mexico; 4 Instituto de Neuroetologia, Universidad Veracruzana. Xalapa, Veracruz, Mexico; 5 Facultad de Medicina. Universidad Autonoma de Nuevo Leon. Monterrey, Nuevo Leon, Mexico

**Keywords:** Cell death, Dopaminergic neurons, Inflammation, Survival, Therapeutics

## Abstract

**Objective(s):**

Parkinson’s disease (PD) is characterized by motor and cognitive dysfunctions. The progressive degeneration of dopamine-producing neurons that are present in the *substantia nigra pars compacta* (SNpc) has been the main focus of study and PD therapies since ages.

**Materials and Methods:**

In this manuscript, a systematic revision of experimental and clinical evidence of PD-associated cell process was conducted.

**Results:**

Classically, the damage in the dopaminergic neuronal circuits of SNpc is favored by reactive oxidative/nitrosative stress, leading to cell death. Interestingly, the therapy for PD has only focused on avoiding the symptom progression but not in finding a complete reversion of the disease. Recent evidence suggests that the renin-angiotensin system imbalance and neuroinflammation are the main keys in the progression of experimental PD.

**Conclusion:**

The progression of neurodegeneration in SNpc is due to the complex interaction of multiple processes. In this review, we analyzed the main contribution of four cellular processes and discussed in the perspective of novel experimental approaches.

## Introduction

Parkinson’s disease (PD) is the second most common neurodegenerative disease worldwide, with high annual costs of treatment (1). This progressive neurological disorder is characterized by gradual progression of neuronal damage in various motor and non-motor circuits (2). Currently, PD affects the adult population (˃65 years) and even young people (3). PD affects a wide variety of nuclei in the central nervous system (CNS), including the dorsal motor nucleus of the vagus, raphe nuclei, *locus coeruleus*, pontine peduncle nucleus, retrorubral nucleus, parabrachial nucleus, the ventral tegmental area, *substantia nigra pars compacta *(SNpc), and *substantia nigra pars reticulata* (SNpr) (4). The degenerative process develops mainly in the dopaminergic neurons (DN), which exhibit native susceptibility to degeneration (5). In humans and the experimental models of PD, the loss of dopaminergic neurons from the SNpc drastically reduces the striatal dopamine concentration (6, 7) promoting motor imbalance, the main characteristic feature that is explored in clinical treatments. 

**Conventional therapies for Parkinson’s disease**

Since years ago, the most commonly used PD treatments has included surgical methods like- pallidotomy or deep brain stimulation (DBS) and pharmacological therapy for each and every PD symptom (8-10). DBS is good at reducing the neuronal loss, avoiding motor fluctuations and preventing damage to the adjacent neurons. On the other hand, DBS is expensive, may cause akinesia and dyskinesia and presence of high risk due to surgical intervention. 

Pharmacological therapy with levodopa (11-13) is specific to the dopaminergic system and decreases motor symptoms; however, it promotes hypersensibility of receptors and overdoses induce dyskinesia. On the other hand, adenosine A2A (14) decreases dyskinesia, inducing low neuroinflammation, but sleep disorders and anxiety are reported. Oral administration of monoamine oxidase type B (MAO-B) inhibitors (15-17) decreases motor disability, prevents the production of free radicals and increases the levels of trophic factors in neurons. However it is not specific for the dopaminergic system, and long-term use may lead to hypertensive crisis, cerebrovascular accident, and weight gain. The oral or subcutaneous use of dopamine agonists (18, 19) lead to neuroprotection of the nigrostriatal pathway, but hallucinations, edema, and addiction have been reported as adverse effects. 

The effectiveness of both pharmacological and gene therapy treatments depends on the level of brain neurodegeneration, and thus, determination of cellular processes at neurodegeneration is the key to improving the treatment effectivity.

**Cellular process associated with degeneration in** ***substantia nigra pars compacta***

***Oxidative stress***

Many scientific reports have demonstrated that oxidative stress produces neurodegeneration (20, 21). In normal conditions of the cell, the reactive oxygen (ROS) and nitrogen (RNS) species act as secondary messengers in cell processes, however, an excess of ROS is responsible for cell degeneration (22, 23). Dopaminergic neurons of SNpc are more susceptible to oxidative/nitrosative damage because they have low levels of glutathione peroxidase and vitamin E; as well as high levels of free iron (pro-oxidant), monoamine oxidase, and neuromelanin (5, 24, 25), for this the intracellular accumulation of ROS can induce mitochondrial respiratory chain blocking, increase of glutamate, and stimulation of NMDA receptors (4) to finally produce excitotoxicity (26) and cell death by necrosis and apoptosis (27). Additionally, in PD animal models has been shown that complex axonal arborization, elevated mitochondrial bioenergetics (28-30), and selective vulnerability of neuronal populations (31) could contribute to the speed of progression of neurodegeneration. Reverting the damage might be possible by controlling or modifying the ROS/RNS, which is one possible key for PD therapy.

***Cell death***

PD is characterized by programmed cell death, which is a homeostatic regulatory function of cells that requires energy in the form of ATP. This programmed cell death is of three types: type I cell death or apoptosis, type II cell death or autophagic cell death, and type III cell death or cytoplasmic cell death (32). In all three types the imbalance of mitochondrial bioenergetics favors DN degeneration in PD (33), which results in alterations of genes such as *alpha-synuclein*, *SNCA, PINK 1, DJ-1, LRRK2, ATP13A2, PLA2G6, FBX07, *and* VPS35* (34-36). In experimental studies three cell death types associated with DN damage have been identified, which include mitophagy (37, 38), autophagy (39, 40), and caspase-3-related apoptosis. Cellular stress can induce activation of caspase-3 by extrinsic and intrinsic pathways of apoptosis in the SNpc (41, 42) and favor the expression of pro-apoptotic genes such as *Bax* and *Bad *similar to ischemic stroke (43). In experimental models, it has linked the role of caspase-3, glycogen synthase kinase 3-beta (GSK3β) and protein kinase Cδ (PK Cδ) as a switch between neurodegeneration and regeneration (42, 44, 45). As apoptosis is the most reported, development of new drugs that could modulate the pathways and direct towards neuronal survival would be one possible key for PD therapy.

***Neuroinflammation***

As per Grunewald *et al*. (37), most studies exhibit the neurons as protagonists in PD. However, the participation of other brain populations gives evidence of a complex phenomenon. The neuroinflammation in PD is also characterized by the presence of increased number of activated microglia and astrocytes around the degenerated neurons (46).

Under high oxidative stress conditions, microglial cells release reactive oxygen / nitrogen species (H_2_O_2_, -NO_3_) and pro-inflammatory cytokines (IL-1β, IL-6 and TNFα) (47), which serve as signals for the recruitment of more microglial cells, causing imbalance in both neuronal growth and in the release of neurotrophic factors (47). The microglia populations present in damaged SNpc can correspond to two opposite types of microglia, cytotoxic (M1 type) and neuroprotective (M2 type) (48). In experimental models of PD, the cytotoxic microglia (M1) have been evidenced during the progress of DN degeneration in SNpc as a consequence of ROS increase, Lewy bodies (LB) formation, and cell death; stimuli as aggregated alpha-synuclein in Lewy bodies may activate M1-microglia and favor the release of pro-inflammatory responses. 

In human post-mortem samples, the alpha-synuclein protein, the main component of LB, has been found in the pre-synaptic terminals of neurons and axons (49). Based on the presence of LB three phases of degenerative damage have been described: 1) LB positive (LB+) neurons without microglia involvement, 2) LB+ neurons with recruited microglia, and 3) LB+ neurons with activated astrocytes. For treating PD the knowledge of the stage-specific switching of M1/M2 phenotypes could be used in therapeutic approaches (48, 50-52).

On the other hand, after neuronal injury, mature astrocytes proliferate and acquire stem cell properties (53-55) promoting neuronal regeneration by synthesizing neurotrophic factors such as glia-derived neurotrophic factor (GDNF) (56) and cerebral dopamine neurotrophic factor (CDNF) (57), and recovery of brain blood irrigation *via* angiotensin type 2 (AT2) (58), the most important effector peptide of the renin-angiotensin system (RAS) (59). Finding a drug that could induce any of the glia to produce more neurotrophic factor or to release anti-inflammatory cytokine production will be a possible key for PD therapy.

***Renin-angiotensin 2 system (RAS)***

The actions of angiotensin 2 (AT2) are mediated by AT1 and AT2-receptors. AT2 increases the differentiation of precursor cells in dopaminergic neurons *via* activation of AT2-receptor (60, 61). It has also been observed that activation of AT2-receptor may inhibit the production of NADPH oxidase (62), supporting the neuroprotective effect due to RAS. However, the overproduction of AT2 could induce inflammation by promoting oxidative stress derived from NADPH *via* AT1-receptors (63, 64), which proposes the amplifying effect of AT2 during dopaminergic degeneration (6, 62). Interestingly, in PD patients increased local and peripheral levels of angiotensin are associated with motor and non-motor symptoms (59, 65-69). 

In experimental models of PD, the high levels of AT2 and ROS induce increased neuron/glial type 2 (NG2) populations (70, 71), precursor cells of immature neurons, oligodendrocytes, Bergmann glia, microglia, and astrocytes depending on the stimulus (46, 57, 72-74). NG2 cells respond very quickly after injury by upregulating the expression of contains chondroitin sulfate proteoglycan 4 (CSPG4) on their surface and exhibit migration and proliferative potential (75-78). Actually, there are no clinical trials evaluating the effect of RAS. Developing or finding a drug that could stimulate the conversion of NG2 cells to immature neurons would be another possible key for PD therapy.

**Novel experimental approaches**

**Figure 1:**
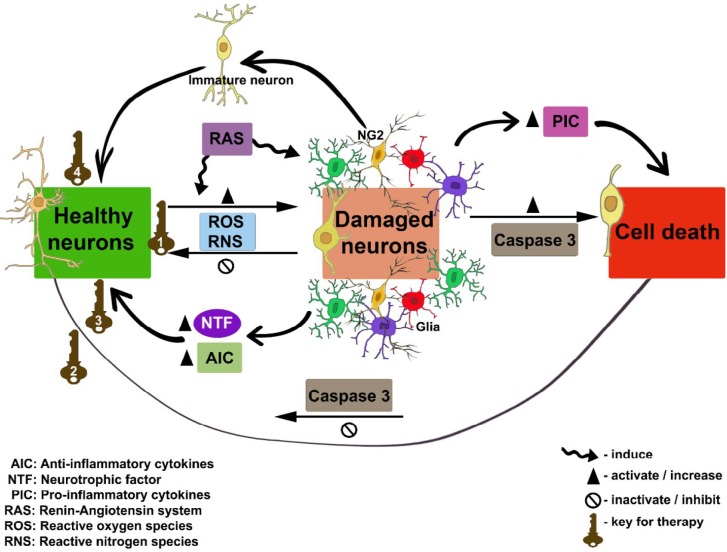
Flow chart of neurodegeneration and hot points for Parkinson’s disease therapy

As oxidative stress, cell death, neuroinflammation, and RAS system play crucial roles in the degeneration process, new drugs that could control or completely revert stress factors might act as keys for PD therapy (79). The Figure 1 shows the interaction of cellular processes above-revised, the new experimental approaches are focused on some of these hot points. Alternative experimental therapies such as targeted gene delivery, specific drugs, and plant-based anti-oxidant approaches are revised.In animal models, focusing the regulation of cell death, the use of GSK3 inhibitors and the upregulation of chaperone-mediated autophagy (CMA) by retinoic acid derivatives and micro RNAs (miRNAs) have yielded discrete results. The disadvantages of GSK3 inhibitors include the inhibition of kinase leading to severe side-effects due to its multiple cellular targets (44); while the upregulation of CMA could be promising by the use of safety administration route (39). Coupled with this, the use of melatonin as a neuroprotective agent continues to be evaluated (80, 81). In the field of control of ROS and neuroinflammation, pretreatment with synthetic neuromodulators (82), curcumin (83), or other plants derivatives (84, 85) could represent benefits, but further studies on bioavailability, dosage, and biosecurity will be required. 

In clinical trials, the capability of GDNF and neurturin to rescue dopaminergic neurons in SNpc (86) has been tested, the results are promising, but due to the lack of safety and specificity, they did not turn out to be a therapeutic medicine (86, 87). In general, targeted gene delivery using viral vectors shows selectivity for dopaminergic neurons, averts neuronal loss, and local increase in the levels of neurotrophic factors that are produced by neurons and glial cells. Unfortunately, currently, these types of strategies are expensive and require biosafety and must be regulated by turn on/off nanosystems expression.

## Conclusion

The multifactorial nature of PD reflects the complex interaction of various cellular processes. The advance in the knowledge of the origin and impact of each related process (stress, neuroinflammation, and cell death) will allow us to better understand the degenerative process and consequently, progress in finding new therapeutic approaches.
